# Self-rated health in rural Appalachia: health perceptions are incongruent with health status and health behaviors

**DOI:** 10.1186/1471-2458-11-229

**Published:** 2011-04-13

**Authors:** Brian N Griffith, Gretchen D Lovett, Donald N Pyle, Wayne C Miller

**Affiliations:** 1 Department of Biomedical Sciences, West Virginia School of Osteopathic Medicine, 400 North Lee Street, Lewisburg, WV 24901, USA; 2 Department of Clinical Sciences, West Virginia School of Osteopathic Medicine, 400 North Lee Street, Lewisburg, WV 24901, USA

## Abstract

**Background:**

Appalachia is characterized by poor health behaviors, poor health status, and health disparities. Recent interventions have not demonstrated much success in improving health status or reducing health disparities in the Appalachian region. Since one's perception of personal health precedes his or her health behaviors, the purpose of this project was to evaluate the self-rated health of Appalachian adults in relation to objective health status and current health behaviors.

**Methods:**

Appalachian adults (n = 1,576) were surveyed regarding health behaviors - soda consumer (drink ≥ 355 ml/d), or non-consumer (drink < 355 ml/d), fast food consumer (eating fast food ≥ 3 times/wk) or healthy food consumer (eating fast food < 3 times/wk), smoking (smoker or non-smoker), exercise (exerciser > 30 min > 1 d/wk) and sedentary (exercise < 30 min 1 d/wk), blood pressure medication (yes, no), and self-rated health. Blood pressure was measured through auscultation and serum cholesterol measured via needle prick. Weight status was based on BMI: normal weight (NW ≥ 18.5 and < 25.0), overweight (OW ≥ 25.0 and < 30.0), and obese (OB ≥ 30.0). Jaccard Binary Similarity coefficients, odds ratios, chi-square, and prevalence ratios were calculated to evaluate the relationships among self-rated health, objective health status, and health behaviors. Significance was set at p < 0.05.

**Results:**

Respondents reported being healthy, while being sedentary (65%), hypertensive (76%), overweight (73%), or hyperlipidemic (79%). Between 57% and 66% of the respondents who considered themselves healthy had at least two disease conditions or poor health behaviors. Jaccard Binary Similarity coefficients and odds ratios showed the probability of reporting being healthy when having a disease condition or poor health behavior was high.

**Conclusions:**

The association between self-rated health and poor health indicators in Appalachian adults is distorted. The public health challenge is to formulate messages and programs about health and health needs which take into account the current distortion about health in Appalachia and the cultural context in which this distortion was shaped.

## Background

The formation of the Appalachian Regional Commission, in 1965, demarcated Appalachia as a specific geographical region consisting of 420 counties in 13 states, where 42% of the population is rural. West Virginia (WV) is mostly rural with 67% of its population living in rural areas. The entirety of WV lies in the heart of Appalachia, a region characterized by poor health and health disparities. For example, among the 50 states, WV ranks between 1 and 3 for obesity, smoking, cancer deaths, cardiovascular disease, stroke, hyperlipidemia, hypertension, heart attacks, and diabetes [1]. WV ranks at the top of the U.S. for poor physical health days (days in previous 30 days), preventable hospitalizations, and the percentage of non-elderly adults limited in activity because of health problems [1,2]. With regard to health behaviors, WV ranks 42^nd ^for fruit and vegetable consumption and 48^th ^for physical activity [1]. Appalachian WV is clearly one of the unhealthiest regions in the country.

Recognizing that many of the health concerns of rural Appalachia are modifiable, numerous health programs and initiatives have been instituted throughout Appalachia over the past several years. A computer search for the Appalachian states reveals numerous programs and interventions targeting health behaviors such as diet and physical activity. Undoubtedly, the intended outcome of these programs and interventions is a reduction in health disparities through individual behavior change.

An underlying theme in most of the major behavior change models is that a person needs to have a realistic perception of the behavioral issue (in this case health behavior) in order to make a successful behavioral change [3]. For example, the Knowledge-Attitude-Behavior model contends that some level of knowledge is prerequisite to the formation of healthier attitudes, and that the formation of new attitudes toward health will result in healthy behavior change [3]. The Behavior Learning Theory asserts that behavior is the outcome of a costs-to-benefits evaluation [3]. Applying this theory to the health behavior exercise, this means that a person will exercise if he/she perceives the benefits of exercise to outweigh the costs. Constructs of the Health Belief Model (HBM) include a person's perceived susceptibility for contracting a disease, the perceived impact of the disease, the perceived benefits in taking action to reduce the threat of contracting the disease, perceived barriers to the preventive behavior, cues to action, and self-efficacy [3]. The primary motivation to change behavior in the HBM is the magnitude of perceived threat of a disease or condition. The primary motivational factor in the Social Cognitive Theory is also governed by perception, the desire to achieve positive outcomes and avoid negative ones [3].

Perception is also the foundation of two of the most popular theories of behavior change - the Theory of Planned Behavior (TPB) and the Transtheoretical Model (stages of change). The TPB argues that behavior is directed by one's attitude toward the behavior in question, the perceived social pressure to perform that behavior (subjective norm), and the ease with which one can perform the behavior (perceived behavioral control) [4]. The combination of a positive attitude, a favorable subjective norm, and a high level of perceived behavioral control lead to a strong intention to perform the behavior. Similar to the TPB, perception is deeply rooted in the Transtheoretical Model (TM), only presented with different constructs. The TM proposes that a person makes a successful behavior change by progressing through certain stages of change [5]. The first two stages, pre-contemplation and contemplation, rely totally on whether the person perceives or does not perceive that there is a problem (or need for behavioral change).

Thus, it seems like, whichever philosophical model for health behavior one favors, the underlying theme for behavior change is individual perception; whether it be perception about the issue (attitude), perceived benefits/costs for performing the behavior, perceived risks of disease, perceived impact of disease, perceived social pressure to participate in the health behavior (subjective norm), perceived control of the health behavior, or the perception of whether or not there is a health issue in the first place. Regardless of whether one embraces the TM or not, one must recognize that the first step prior to behavior change in any theoretical model is the perception that there is an issue. If a person does not perceive health to be an issue (pre-contemplation), then he/she will never progress through the stages of change in the TM. Similarly, if a person does not perceive health to be an issue, he/she will not acquire knowledge or attitudes to change behavior in the Knowledge-Attitude-Behavior model, evaluate costs and benefits of behavior change in the Behavior Learning Theory, form perceptions necessary for change in the HBM, develop perceived behavioral control in the TPB, etc.

Consequently, one's perception of health, or one's self-rated health, is the antecedent to his/her entering the pathway to health behavior change. It seems logical, then, that the first step in helping the residents of Appalachia improve their health status is to evaluate their self-rated health. If Appalachians perceive themselves to be healthy, then interventions targeting health behavior change are going to be ineffective. On the other hand, if Appalachians perceive themselves as being unhealthy, then health behavior interventions are more likely to be successful. The purpose of this project, therefore, was to evaluate the self-rated health of Appalachian adults in relation to their objective health status and current health behaviors.

## Methods

Self-rated health status, objective health status, and health behaviors were evaluated in Appalachian adults in order to determine if ratings of perceived health were consistent with objective health status and health behaviors. Variables of interest were perceived health status, blood pressure, use of blood pressure medications, serum cholesterol, obesity, smoking, fast food consumption, regular soda consumption, and exercise. The study procedures were approved by the West Virginia School of Osteopathic Medicine Institutional Review Board for the Protection of Human Research Participants.

### Sample Population

**Figure 1:**
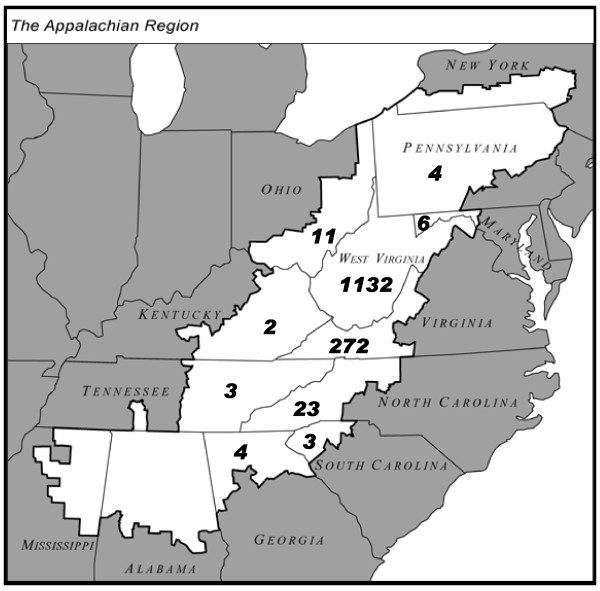
**The Appalachian region**. The Appalachian Region as defined by the Appalachian Regional Commission is highlighted in white. The numbers in each Appalachian state represent the number of Appalachian participants from that state who participated in the survey.

The sample population consisted of 1,576 adults, aged 19-92 y, from all areas of rural Appalachia, who attended a WV State Fair blood pressure booth, conducted in Greenbrier County, WV (Figure 1). Initially, 1988 participants volunteered for the study, but 412 were excluded from data analysis based on age (<18 y), missing self-reported anthropometric data, or partially completed surveys. A small subset of the sample volunteered for an on-site cholesterol screening (n = 91). Although only a small percentage of the sample population received the cholesterol screening, this sub-group was representative of the entire sample. Analysis of demographic data and response patterns of those who received the cholesterol screening revealed that they were similar to those who did not receive cholesterol screening for average age, BMI, and blood pressure. In addition, the same percentage of this sub-group reported being male, being healthy, smoking, exercising, consuming fast food, drinking regular soda, and at the same income level as those not tested for cholesterol.

### Procedures

Participants in the study were given a survey containing 10 questions regarding gender, age, height, weight, smoking, nutrition behaviors, exercise behavior, blood pressure, use of blood pressure medication, cholesterol levels, and self-rated health. Body weight and height were self-reported and were used to calculate body mass index (BMI). Self-reported height and weight have been shown to be valid and reliable [6,7]. A classification of normal weight (NW), overweight (OW), and obese (OB) were assigned based on BMI stratification (NW ≥ 18.5 and < 25.0, OW ≥ 25.0 and < 30.0, OB ≥ 30.0). Smoking was evaluated by responses to the question, "Do you currently smoke, yes or no." Nutrition behaviors were assessed by two variables, regular soda consumption and fast food consumption. Regular soda was understood to be a non-alcoholic sugar-sweetened carbonated drink. Regular sodas in the U.S. contain approximately 40 g of sugar and 150 kcal per 355 ml can. Drinking regular soda was dichotomized as a soda consumer (drink ≥ 355 ml soda per d) or non-consumer (drink < 355 ml soda per d) [8]. Fast food consumption was dichotomized as fast food consumer (eating fast food 3 or more times a wk) and healthy food consumer (eating fast food < 3 times a wk) [9]. Exercise participation was dichotomized as sedentary (exercise ≤ 1 day per week for < 30 min) or active (exercise > 1 day per week for ≥ 30 min).

Blood pressure was measured manually, through auscultation. All blood pressure measurements were taken by a second year medical student, a staff nurse, or a phlebotomist from the West Virginia School of Osteopathic Medicine; who were under the supervision of a physician. Although only one blood pressure measurement is not suitable for a medical diagnosis, for the purpose of this study, hypertension was defined as having a systolic blood pressure ≥ 140 mm Hg or having a diastolic blood pressure ≥ 90 mm Hg. Use of blood pressure medication was assessed by answering the question, "Do you currently take any blood pressure medication, yes or no." Self-rated health was determined by answering the question, "In general, would you say your overall health right now is excellent, good, fair, or poor." A self-rating of "healthy" was defined as reporting either excellent or good health. In other populations, self-rated health has been shown to be an accurate reflection of a person's health, a valid predictor of chronic morbidity, and a quality indicator for primary health care in rural populations [10-13].

Total cholesterol was measured using the Cholestech L.D.X. system according to the manufacturer's instructions (Colestech, Hayward, CA). Participants who volunteered for the cholesterol screening gave a blood sample and were given the results of the cholesterol screen prior to answering the questionnaire. Consequently, participants had their cholesterol numbers to report prior to self-rating their health status.

### Statistical Analyses

All statistical analyses were performed using the Systat 12 statistical software package (Systat Software, Inc.; Chicago, IL). Empty data cells were not included in the analyses. Group data are reported as mean ± SEM. Jaccard Binary Similarity Coefficients were calculated to determine the proportion of times two events occur, given at least one event occurs. For example: if somebody reported being either healthy or obese, what was the chance that they reported being both healthy and obese? Two-by-two tables of frequency of responses were constructed for dichotomous variables. Subsequently, the Pearson chi-square test was used to assess for independence of row and column variables. Odds ratios, with 95% confidence intervals, were also calculated for the same variables. Statistical significance was declared at the *P *< 0.05 level.

## Results

The demographics of the sample population are presented in Table 1. A large portion of the respondents (74%) considered themselves healthy. Age did not modify perception of health, in that 75% of those ≤ 40 y considered themselves healthy, 75% of those 41-60 y considered themselves healthy, and 73% of those > 60 y believed they were healthy. A Chi-Square test of association for age and self-rated health showed no significant relationship (p = 0.235). While a large portion of the respondents considered themselves healthy, 65% were sedentary, 24% were hypertensive, and 73% obese. Although the sample population was not randomly selected from the entire Appalachian region, the demographics of the state of WV (which is entirely Appalachian) are almost identical to the study sample for being sedentary, hypertensive, overweight or obese, and hypercholesterolemic. Thus, the study sample seems to be a good representation of the Appalachian region.

**Table 1:** Participant demographics

	Men (n = 770)	Women (n = 804)	All (n = 1576)
Age	54.4 ± .5	53.1 ± .5	53.8 ± .4
Height (cm)	178.5 ± .2	163.5 ± .2*	170.8 ± .3
Weight (kg)	91.9 ± .6	75.6 ± .6*	83.5 ± .5
BMI	28.7 ± .2	28.2 ± .2*	28.5 ± .1

Disease, Symptom, or Behavior	Number (%)	Number (%)	Number (%)

Healthy	530 (74)	573 (75)	1105 (74)
Unhealthy	188 (26)	195 (25)	383 (26)
Smoker	70 (9)	88 (11)	158(10)
Non-Smoker	694 (91)	714 (89)	1408 (90)
Fast Food Consumer	208 (27)	145 (18)	353 (22)
Non-Fast Food Consumer	562(73)	659 (82)	1221 (78)
Soda Drinker	524 (68)	523 (65)	1047 (67)
Non-Soda Drinker	246 (32)	281 (35)	527(33)
Exercise	252 (33)	297 (37)	549 (35)
Sedentary	518 (67)	507 (63)	1025 (65)
Hypertensive	227 (29)	157 (20)	384 (24)
Normo-tensive	543 (71)	647 (80)	1190 (76)
Blood Pressure Medication	320 (44)	308 (40)	628 (42)
No Blood Pressure Medication	404 (56)	468 (60)	872 (58)
Normal Weight	155 (20)	274 (34)	429 (27)
Overweight	354 (46)	264 (33)	618 (39)
Obese	261 (34)	266 (33)	527 (34)
Overweight or Obese	615 (80)	530 (66)	1145 (73)
Normal Cholesterol	38 (64)	19 (53)	57 (60)
High Cholesterol	21 (36)	17 (47)	38 (40)

*Significant difference between men and women.

Table 2 shows the proportion of respondents who considered themselves healthy in spite of the fact that they had a disease or disease symptom, and/or were participating in poor health behaviors. Between 57% and 79% of the respondents who considered themselves healthy had at least one disease condition, symptom or poor health behavior. The Jaccard Binary Similarity coefficients for the pairing of perceived health with disease, disease symptoms or poor health behaviors also revealed a high likelihood of a respondent reporting being healthy, when he/she had a disease, disease symptom or poor health behavior (Table 3). The analyses in Table 4 show that the odds of a person who has a disease, disease symptom, or poor health behavior perceiving him or herself as healthy is higher than an expected odds ratio of zero, for comparisons to those who don't have a disease, disease symptoms, or poor health behaviors and perceive themselves to be healthy. Odds ratios for perceived health in the objectively healthy versus the objectively unhealthy were equivalent for high cholesterol and fast food consumption.

**Table 2:** Percentage of Appalachian adults reporting being healthy with manifestation of disease, disease symptom or poor health behavior

Disease, Symptom, or behavior															
Healthy Self Rating	X	X	X	X	X	X	X	X	X	X	X	X	X	X	X
Sedentary	X									X				X	X
Fast Food Consumer		X								X				X	X
Soda Drinker			X							X				X	X
Smoker				X											
Hypertensive					X						X	X	X		X
Blood Pressure Medication						X					X	X	X		X
Obese							X					X	X	X	X
Overweight or Obese								X							
High Cholesterol									X				X		
% of Respondents	65	72	67	65	64	65	58	66	79	66	65	57	63	64	65

**Table 3:** Jaccard binary similarity coefficients for perceived health and manifestation of disease, disease symptom or poor health behavior in Appalachian adults

Disease, Symptom or behavior	Healthy
Sedentary	0.28
Fast Food Consumer	0.20
Soda Drinker	0.51
Smoker	0.09
Hypertensive	0.20
Blood Pressure Medication	0.31
Obese	0.23
Overweight or Obese	0.53
High Cholesterol	0.32

**Table 4:** Odds of the perception of being healthy and having a disease, disease symptom, or poor health behavior in Appalachian adults

Disease, Symptom, or behavior	O.R.	95% C.I.	O.R. *P*-value	Chi-square *P*-value
Sedentary	0.61	-0.73 to -0.26	0.001	0.001
Fast Food Consumer	0.80	-0.49 to 0.05	0.110	0.110
Soda Drinker	0.65	-0.69 to -0.18	0.001	0.001
Smoker	0.65	-0.79 to -0.07	0.018	0.017
Hypertensive	0.69	-0.64 to -0.12	0.005	0.005
Blood Pressure Medication	0.48	-0.97 to -0.50	0.001	0.001
Obese	0.37	-1.24 to -0.76	0.001	0.001
Overweight or Obese	0.44	-1.13 to -0.53	0.001	0.001
High Cholesterol	1.11	-0.89 to 1.09	0.840	0.840

## Discussion

The purpose of this project was to evaluate the self-rated health of Appalachian adults in relation to their objective health status and current health behaviors. Overall, 74% of the Appalachians surveyed believed they were in good health. This finding is contradictory to what is known about Appalachian health. It is well established that the Appalachian region is one of the unhealthiest regions in the country [1,2]. Moreover, this current sample population, which perceived themselves as being healthy, demonstrated a high prevalence of hypertension, obesity, inactivity, and poor nutrition (Table 1). Thus, objective health measures in Appalachian adults did not correspond with their rating of perceived health.

This disconnect between perceived health and morbidity is generally not seen in other populations [10-17]. Most often, there is a high correlation between self-rated health and morbidity, to the extent that the risk of mortality is sometimes more strongly associated with self-rated health than with objective health status [14]. Further evidence of the disconnect between self-rated health and objective health status in Appalachia was seen when the positive self-rated health responses to objective health status measures and health behaviors of the participants were matched (Tables 2-3). Between 58% and 79% of respondents who reported being healthy had at least one indicator of poor health (Table 2). It is even more interesting to note that between 57% and 66% of participants who reported being healthy had multiple conditions or behaviors that would indicate otherwise. Probably the most stunning result is that 65% of those who were on blood pressure medication, but still had elevated blood pressure, reported being healthy.

The pattern seen in which objectively unhealthy Appalachians report that they are healthy is reinforced with the calculation of the Jaccard binary similarity coefficients for self-reported health status and the unhealthy conditions or behaviors (Table 3). Although the Jaccard coefficients do not appear to be high (if one were to erroneously compare the Jaccard coefficients to common correlation coefficients), the interpretation of the Jaccard coefficients is quite noteworthy. Since the Jaccard coefficient gives the proportion of times both events occur, given at least one occurs; an example of the interpretation is as follows: if somebody reported being either healthy or having high cholesterol, the chance of them reporting that they were both healthy and hyperlipidemic is 32%. The interpretation of these Jaccard coefficients becomes meaningful in that a significant number of Appalachian adults do not properly associate their health status with their health condition or behavior. For example, one out of two Appalachians who are overweight or obese would say that they are healthy; and one out of two people who would say that they are healthy are overweight or obese.

Odds ratios for a person with a poor health indicator reporting being healthy compared to those without a poor health indicator reporting being healthy vary somewhat in magnitude (Table 4). However, the odds ratios are relatively high, considering one would not expect people with poor health indicators to see themselves as healthy. The odds ratios for perceived health for those people with high cholesterol were equivalent to those who had normal cholesterol levels, meaning that knowledge of hyperlipidemia in these Appalachian adults had no effect on their perceived health. The same held true for fast food consumption. These inter-person comparisons (odds ratios) parallel the findings of the previously highlighted intra-person comparisons (pair wise comparisons and Jaccard coefficients). The conclusive analysis is that Appalachian adults do not associate objective health indicators with perceived health.

The impact of the dissociation between self-rated health status and poor health indicators in Appalachia is demographically apparent. For example, more than 70% of Appalachia has the highest prevalence of obesity in the US, with areas of KY, TN, and WV having 80% of their population with obesity or its common co-morbidity, type 2 diabetes [18]. Despite state and federal policies targeting this epidemic, the problems of obesity and diabetes continue to plague the region, with no signs of reducing this trend. Our research suggests that one reason for limited success in fighting health disparities in Appalachia is based on distorted perceptions of health. We found a high percentage of people who were clinically identified as obese, hypertensive, and hyperlipidemic classifying themselves as in good health. We also found that a high percentage of people with poor eating and exercise behaviors classified themselves as healthy.

From the standpoint of health risks or behaviors leading to health risks, the results from this study may fall in line with what Manderbacka et al. found several years ago [19]. They found no association between dietary fat avoidance and self-related health in Swedish adults; a finding that would parallel the Appalachians' dissociation between frequency of fast food consumption and self-rated health. These same investigators found an initial association between leisure time physical activity and self-rated health, but when health problems and functional limitations were controlled for, the association disappeared. Similarly, the association between BMI and self-rated health disappeared when controlling for health problems and functional limitations. The authors conclude that risk factors and health behaviors are not considered directly when assessing one's own health, but their potential effect on self-rated health is mainly mediated by more specific health problems and their functional consequences [19].

This may be the case with rural Appalachians. Obesity, hypertension, and hyperlipidemia may not be considered health issues in Appalachia until these conditions cause functional limitations. Similarly, health risk behaviors like fast food consumption, soda drinking, and a sedentary lifestyle may not be associated with poor health until a direct personal link between these behaviors and functional limitations has been established in the mind of the individual. This theory is further supported by the known fact that although the Appalachian region is one of the least healthy in the country, frequency of visits to health care providers is disproportionately low [20]. It appears that interventions focused on improving health behaviors in rural Appalachia are not going to be successful until the rural Appalachian's health perception becomes congruent with his/her health risk behaviors and objective measures of health.

Previously established poor health indicators, along with the currently established distorted perceptions of health, then, must be seen as a cultural backdrop for future interventions in Appalachia. The participants in this study were those who were able to leave their home, make it to the WV State Fair and either self-ambulate about the premises or use assisted ambulation devices. At any rate, they came from families and communities in which the "common state of affairs" is to be hypertensive, hyperlipidemic, sedentary, and overweight or obese. Furthermore, typical eating and drinking patterns in their home communities were likely to normalize frequent fast food and soda consumption. Thus, participants responded to a survey about their own health in this community context. Compared to the people with whom they are familiar (e.g., their family members, co-workers, neighbors, and community members) they are relatively healthy. Thus, dichotomous notions of health versus the alternative will not appropriately motivate the Appalachian individual to change his or her health behaviors. Education, therefore, must be targeted at promoting appropriate views of health and the need for improved health.

## Conclusions

High priorities for programs targeting health disparities in rural Appalachia should be those that target one's perception of health and/or one's perception of health risk behaviors. Moreover, public health programs targeting positive health behaviors may be more readily received than those targeting negative behaviors [21,22]. For example, Dave et al. [21] suggest that public education regarding the unhealthiness of fast food may not influence fast food consumption, but that education targeting the issue of convenience and quick or efficient preparation of nutritious alternatives to fast food may be more promising. Similarly, public messages about quick and easy ways to incorporate physical activity into one's current lifestyle may be received better than messages attacking the health risks of a sedentary lifestyle. Messages about how lifestyle changes to control body weight, hypertension, and hyperlipidemia will improve functional capacity and increase vigor and vitality might be better received than messages about how not controlling these conditions can lead to sickness, lethargy and early mortality.

It should also be remembered that the formation and delivery of new public messages and programs for rural Appalachians should be focused on people who are unhealthy and have poor health behaviors, but believe they are healthy. With regard to behavior change models, this means focusing on moving people through the pre-contemplation or contemplation stage in the TM, focusing on the shaping of positive attitudes in the Knowledge-Attitudes- Behavior model and the TPB model, increasing benefits-to-cost ratios in the Behavior Learning Theory and HBM, increasing positive outcomes in the Social Cognitive Theory, and increasing perceived behavioral control in the TPB - schemas which all refer back to individual perceptions. The challenge, then, becomes formulating messages and programs about health and health needs which take into account the current distortion about health in Appalachia and the cultural context in which this distortion was shaped.

## Competing interests

The authors declare that they have no competing interests.

## Authors' contributions

BNG conceived the idea, designed the study, collected data and assisted with the manuscript preparation. GDL assisted with the study design, collecting data, and with the editing of the manuscript. DNP assisted with data collection and data input. WCM helped modify the study design, ran the statistical analyses, and drafted the manuscript. All authors read and approved the final manuscript.

## Pre-publication history

The pre-publication history for this paper can be accessed here:

http://www.biomedcentral.com/1471-2458/11/229/prepub
